# White matter capillaries in vascular and neurodegenerative dementias

**DOI:** 10.1186/s40478-019-0666-x

**Published:** 2019-02-07

**Authors:** Yoshiki Hase, Ren Ding, Gina Harrison, Emily Hawthorne, Amilia King, Sean Gettings, Charlotte Platten, William Stevenson, Lucinda J. L. Craggs, Raj N. Kalaria

**Affiliations:** Neurovascular Research Group, Institute of Neuroscience, Newcastle University, Campus for Ageing and Vitality, Newcastle upon Tyne, NE4 5PL UK

**Keywords:** Alzheimer’s disease, Dementia, Dementia with Lewy bodies, Microvascular pathology, Mixed dementia, Parkinson’s disease with dementia, Post-stroke dementia, Small vessel disease, Vascular dementia

## Abstract

Previous studies suggest white matter (WM) integrity is vulnerable to chronic hypoperfusion during brain ageing. We assessed ~ 0.7 million capillary profiles in the frontal lobe WM across several dementias comprising Alzheimer’s disease, dementia with Lewy bodies, Parkinson’s disease with dementia, vascular dementia, mixed dementias, post-stroke dementia as well as post-stroke no dementia and similar age ageing and young controls without significant brain pathology. Standard histopathological methods were used to determine microvascular pathology and capillary width and densities in 153 subjects using markers of the basement membrane (collagen IV; COL4) and endothelium (glucose transporter-1; GLUT-1). Variable microvascular pathology including coiled, tortuous, collapsed and degenerated capillaries as well as occasional microaneurysms was present in all dementias. As expected, WM microvascular densities were 20–49% lower than in the overlying cortex. This differential in density between WM and cortex was clearly demonstrated by COL4, which was highly correlated with GLUT-1 densities (Spearman’s rho *=* 0.79, *P* = 0.000). WM COL4 immunopositive microvascular densities were decreased by ~ 18% across the neurodegenerative dementias. However, we found WM COL4 densities were increased by ~ 57% in post-stroke dementia versus ageing and young controls and other dementias. Using three different methods to measure capillary diameters, we found WM capillaries to be significantly wider by 19–45% compared to those in overlying neocortex apparent with both COL4 and GLUT-1. Remarkably, WM capillary widths were increased by ~ 20% across all dementias compared to ageing and young controls (*P* < 0.01). We also noted mean WM pathology scores incorporating myelin loss, arteriolosclerosis and perivascular spacing were correlated with COL4 immunopositive capillary widths (Pearson’s r = 0.71, *P* = 0.032). Our key finding indicates that WM capillaries are wider compared to those in the overlying neocortex in controls but they dilate further during dementia pathogenesis. We suggest capillaries undergo restructuring in the deep WM in different dementias. This reflects compensatory changes to retain WM perfusion and integrity during hypoperfusive states in ageing-related dementias.

## Introduction

The cerebral white matter (WM) has become an important focus for investigation of mechanisms in brain ageing and dementia. Age is the single most important risk factor for WM damage, which relates to increased white matter hyperintensities (WMH) on T2- weighted magnetic resonance imaging (MRI) and associated with vascular disease, disability, cognitive impairment and death [9, 15, 18]. Cerebral hypoperfusion in older age is implicated as a key pathophysiological element, which contributes to diffuse WM changes. Both cerebral small vessel disease and carotid artery disease are risk factors for cerebral WM damage [4, 9]. WM vascular pathology in brain ageing is observed with a frequency of 71% in non-demented versus 84% in demented subjects [27]. WM vascular changes are most prominent in vascular dementia [10]. However, all neurodegenerative dementias bear some degree of vascular pathology ranging from 61% in frontotemporal dementias to 82% in AD [35]. A significant portion of such pathology is attributed to small vessel disease in the WM [10]. Previous imaging and pathological studies indicated that the medullary arteries and WM of the frontal lobe are especially susceptible to haemodynamic derangement, leading to more severe WM damage, than for example in the temporal lobe, during ageing and vascular disease [13, 17]. Obstruction of lumen at the proximal ends of penetrating arteries or becoming coiled or tortuous enroute would reduce blood pressure and impact on perfusion of the WM [6]. Furthermore, age-related arteriosclerotic changes and segmental loss of vascular smooth muscle cells along lengths of both the medullary and perforating arteries disrupting flow in the distal arteries and affecting perfusion of the deep WM with the creation of an hypoxic environment [12, 26].

The microvascular network within the WM though less dense than in the neocortex is equally vulnerable. In ageing rats, primates and man endothelial cells of cortical cerebral microvessels shrink with thickening of the basal lamina [21, 24]. The endothelium is activated as indicated by increased expression of the intercellular adhesion molecule in relation to basement membrane collagen IV (COL4) [12]. The activation is often accompanied by proliferation of microglia, which release proteases and free radicals to promote damage to components of the extracellular matrix that contain high amounts of collagen [32]. The microvascular network undergoes severe distortions including tortuosity, coiling and kinking [6], which increase with age and coincides with leukoaraiosis. Consistent with low tissue oxygen tension, within the deep WM there is induction of hypoxia-inducible factors 1alpha and 2alpha as well as key hypoxia-regulated proteins such as matrix metalloproteinase-7 and neuroglobin [12]. These observations are consistent with elevated concentrations of the vasoconstrictor endothelin 1, reflecting abnormal regulation of WM perfusion [5]. However, it is not clear at what threshold point diffuse WM damage implied by the microvascular changes tips over to affect cognitive function.

In this study, we focused on microvessels of the frontal WM as a key component of the gliovascular unit facilitating tissue perfusion [16]. We concentrated on the frontal lobe because of its contiguity with the centrum semiovale region and relative vulnerability in cerebrovascular disease [20, 21]. We specifically assessed capillary changes in relation to WM damage across various neurocognitive disorders including Alzheimer’s disease (AD), dementia with Lewy bodies (DLB), Parkinson’s disease with dementia (PDD), Vascular dementia (VaD) as well as post-stroke dementia (PSD).

## Materials and methods

### Study design and subjects

The study comprised 153 subjects derived from longitudinal prospective dementia series and ageing controls (Table 1). Dementia was clinically diagnosed and pathologically confirmed by post-mortem examination as either AD, DLB, PDD, mixed DLB, PDD, AD and VaD (who met two or more neuropathological diagnostic criteria for DLB, PDD, AD and VaD) (Mixed 1), mixed AD-VaD (Mixed 2), VaD or PSD. In addition, we compared post-stroke no dementia (PSND) subjects as well as young and ageing controls. The young control subjects aged 55–68 and ageing control subjects aged 72–99 years were either participants in the previous prospective studies [1] or were recruited based on unrelated brain donations to the Newcastle Brain Tissue Resource (NBTR). They were selected to be included as controls if they had not been diagnosed with cognitive impairment or any neurological or psychiatric illness or did not show any signs of neurological disorders. The VaD, PSD and PSND subjects were from the Newcastle Cognitive Function After Stroke study [1]. Local research ethics committees of the Newcastle upon Tyne NHS Foundation Hospitals Trust granted ethical approvals. Permission for use of brains for post-mortem research was also granted by consent from the individuals themselves when they had been still alive or next-of-kin or family member. All the brain tissues were retained and obtained from the NBTR.

**Table 1:** Demographic details, clinical and pathological features in cases used for microvascular quantification

Variable	Young Controls	Ageing Controls	PSND	AD	DLB	PDD	Mixed 1	Mixed 2	VaD	PSD
Number of subjects	9	16	18	18	16	13	17	14	13	19
Age, years, mean (range)	61.1↓(55–68)*	86.9 (72–99)	84.7 (79–91)	87.8 (76–96)	79.6 (69–96)	72.8 (64–81)	83.0 (72–93)	84.7 (72–94)	88.1 (75–98)	87.5 (80–98)
Gender, number (F/M)	5 / 4	12 / 4	9 / 9	9 / 9	7 / 9	5 / 8	13 / 4	8 / 6	6 / 7	13 / 6
Total CAMCOG score (/100), mean (range)	N/A	N/A	88.0↑ (83–93)*	44.0 (20–73)	53.8 (24–80)	61.1 (39–74)	51.0 (23–68)	50.5 (41–61)	62.0 (25–80)	61.5 (24–80)
Memory sub-score (/27), mean ± SEM	N/A	N/A	21.4 ± 1.4↑*	10.0 ± 0.9	14.9 ± 1.1	16.2 ± 1.1	13.5 ± 1.6	N/A	14.4 ± 1.4	15.0 ± 2.2
Executive sub-score (/28), mean ± SEM	N/A	N/A	16.6 ± 1.2↑*	12.3 ± 0.6	12.1 ± 1.5	9.6 ± 0.9	11.2 ± 0.8	N/A	10.8 ± 1.2	11.1 ± 1.9
MMSE score (/30), mean (range)	N/A	N/A	27.3↑ (26–30)*	8.5 (0–16)	13.8 (6–20)	14.2 (3–20)	11.9 (2–19)	14.0 (12–16)	17.3↑ (8–24)^¶^	16.5 (12–20)
Braak Stage, mean (range)	0.25 (0–1)	1.9 (0–4)	2.6 (1–4)	5.6↑ (5–6)**	2.3 (0–4)	2.1 (0–4)	4.9↑ (2–6)**	5.2↑ (5–6)**	2.0 (0–4)	2.6 (1–4)
CERAD, mean (range)	0.0 (0–0)	0.5 (0–2)	1.7 (1–2)	2.9↑ (2–3)**	1.3 (0–3)	0.3 (0–2)	2.7↑ (0–3)**	2.9↑ (2–3)**	1.0 (0–2)	1.3 (1–3)
Vascular pathology score, mean (range)^♯^	N/A	6.7↓ (0–10) ^‡^	13.5 (13–14)^¶^	10.8 (3–16)	9.6 (7–13)	9.8 (7–14)	10.6 (3–13)	11.0 (6–14)	13.2↑ (10–16)^¶^	13.3↑ (9–17)^¶^
WML score, mean (range)	N/A	0.5↓ (0–2)^§^	2.5↑ (2–3)^ψ^	1.7 (0–3)	1.7 (1–3)	1.8 (1–3)	1.6 (0–3)	2.8↑(2–3)^ψ^	2.9↑(2–3)^ψ^	2.4↑(2–3)^ψ^
White matter/Vascular lesions, moderate - severe (%)	N/A	17.6↓**	100	72	75	92	88	95	100	100
Lewy body pathology, number (limbic/neocortical)	0 / 0	0 / 0	0 / 0	0 / 1	3 / 11	3 / 7	3 / 5	0 / 0	4 / 0	0 / 0
Neuronal loss in substantia nigra, number (none/mild/moderate/severe)	N/A	N/A	18 / 0 / 0 / 0	7 / 8 / 2 / 0	0 / 3 / 6 / 6	0 / 1 / 2 / 9	3 / 4 / 7 / 2	14 / 0 / 0 / 0	7 / 4 / 2 / 0	19 / 0 / 0 / 0

Mixed dementia 1: 7 = AD+DLB, 6 = AD+VaD, 3 = AD+DLB + VaD and 1 = PDD + DLB + VaD; Mixed dementia 2: all with AD and VaD pathology

Age, *P < 0.01 vs Ageing Controls and all dementia groups; Total CAMCOG score, CAMCOG executive and memory sub-score and MMSE score, *P < 0.01 vs all dementia groups;

Braak Stage, **P < 0.01 vs Young Controls, Ageing Controls, DLB, PDD and VaD; **P < 0.05 vs PSD and PSND; CERAD, **P < 0.01 vs Young Controls. Ageing Controls, DLB, PDD, VaD, PSD and PSND

♯Vascular Pathology Score [10], ^‡^*P* < 0.01 vs all dementia groups; ^¶^*P* < 0.01 vs DLB and PDD; ^¶^*P* < 0.05 vs Mixed 1; WML score, ^§^*P* < 0.01 vs all dementia groups; ^ψ^*P* < 0.01 vs AD, DLB, PDD and Mixed 1; White matter/Vascular lesions, moderate-severe (%) **P < 0.01 vs all dementia groups; Lewy body pathology, only the number of limbic/neocortical cases are shown. Fourteen Ageing Controls, 15 AD, 6 Mixed 1, all Mixed 2, 9 VaD, all PSD and PSND cases had no Lewy body pathology. Two Ageing Controls, 1 AD, 1 DLB and 2 Mixed 1 showed Lewy body pathology in brain stem. Data were not available for 1 case in DLB and PDD groups due to limited autopsy; Neuronal loss in the substantia nigra, data was not available for 1 case in each group other than VaD, PSD and PSND due to limited autopsy

Abbreviations: *AD* Alzheimer’s disease, *CAMCOG* Cambridge Cognition Examination, *CERAD* Consortium to Establish a Registry for Alzheimer’s Disease, *DLB* Dementia with Lewy Bodies, *F* female, *M* male, Mixed 1, Mixed dementia 1 with AD, DLB, PDD and VaD; Mixed 2, Mixed dementia 2 with AD and VaD, *MMSE* Mini Mental State Examination, *PDD* Parkinson’s disease with dementia, *PSD* post-stroke dementia, *PSND* post-stroke no dementia, *VaD* Vascular dementia, *WML* white matter lesion

### Brain tissues and neuropathological analysis

Neuropathological assessment was carried out as described previously [1, 14]. Briefly, haematoxylin and eosin (H&E) staining was used for assessment of structural integrity and infarcts, Nissl and Luxol Fast blue staining for cellular patterns and myelin loss, Bielschowsky’s silver impregnation and amyloid-β for Consortium to Establish a Registry for Alzheimer’s Disease (CERAD) rating of neuritic plaques, Gallays stain for neuritic pathology, and tau immunohistochemistry for Braak staging of neurofibrillary tangles [21, 22]. The clinical diagnoses of DLB and PDD were confirmed according to established criteria [28]. The clinical diagnosis of AD was confirmed on evidence of significant Alzheimer’s-type pathology, namely a Braak stage V–VI score, a moderate-severe CERAD score [26] and an absence of significant vascular pathology. The clinical diagnosis of vascular dementia (VaD) was made when there were multiple or cystic infarcts, lacunae, border-zone infarcts, microinfarcts and small vessel disease, and pathologically confirmed as Braak stage ≤ IV [21, 22]. Mixed AD and VaD case was classified when there was sufficient degree of pathology to reach Braak V–VI and significant vascular pathology [3]. We also included cases with Mixed dementia, who met two or more neuropathological diagnostic criteria for DLB, PDD, AD and VaD (Mixed 1) and AD-VaD (Mixed 2) (Table 1).

Vascular pathology scores were derived from the presence of vascular lesions/pathologies in four brain areas, including the frontal lobe at the level of the olfactory bulbs, temporal lobe at level of the anterior hippocampus, basal ganglia at level of mammillary body and middle segment of the hippocampus [10]. Lesions including arteriolosclerosis, cerebral amyloid angiopathy, perivascular haemosiderin leakage, perivascular space dilatation in the deep and juxtacortical WM, myelin loss, and cortical micro (< 0.5 cm) and large (> 0.5 cm) infarcts were recorded with increasing severity resulting in greater scores [10]. The relative presence of string and coiled vessels was assessed in a semi-quantitative manner. WM lesion (WML) scores were determined on scale of 0 to 3 signifying none, mild, moderate and severe. Previously, we had shown there was 95% agreement in scoring between two assessors [10]. WM/vascular lesion severity was graded from low to severe in quartiles essentially as described previously [17]. All the vascular measures are compatible with the recently established vascular cognitive impairment neuropathology consortium criteria [34]. Tissues from ageing control subjects had occasional ageing-related pathology and were classified as ‘no pathological diagnosis’ (Table 1). Except for neuropathological examination (RNK), all subsequent morphological analyses were always undertaken under operator-blinded conditions. Samples were identified with coded sequential numbers. In addition, at least two of both positive and negative controls were included to monitor the quality of staining.

### Immunohistochemistry methods

**Fig. 1:**
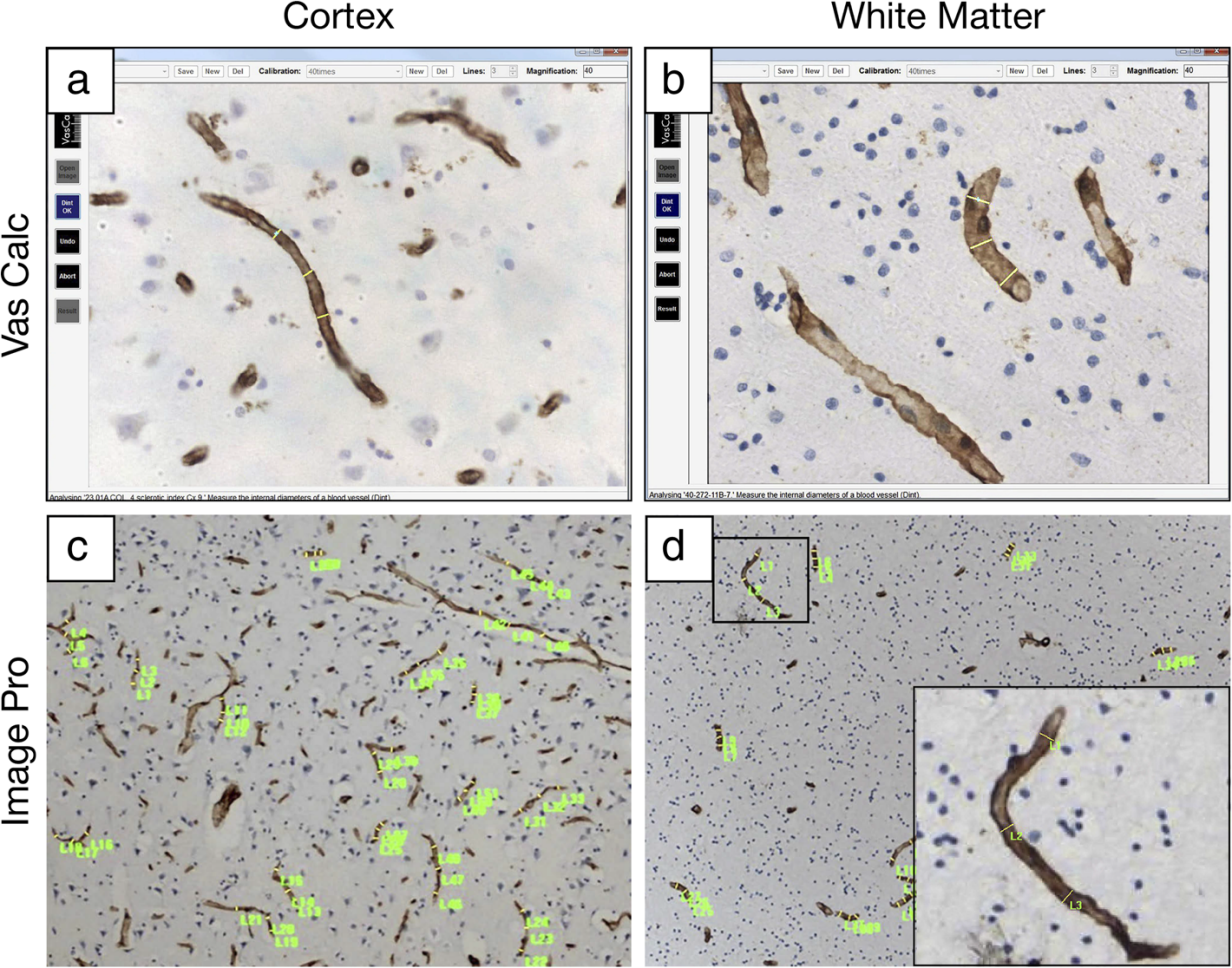
Methods used to quantify microvascular morphology **a**-**d**, Representative images of collagen-IV (COL4) stained microvessels in the cortex (**a**, **c**) and WM (**b**, **d**). **a**-**b**, Screen shots of profiles of capillaries indicating how widths (diameters) were measured longitudinally using the VasCalc method using 40x objective lens. **c**-**d**, Images of capillaries with indications (in green markers) where widths along the vessel were measured using the Image-Pro Analyser method using 10x objective lens

**Fig. 2:**
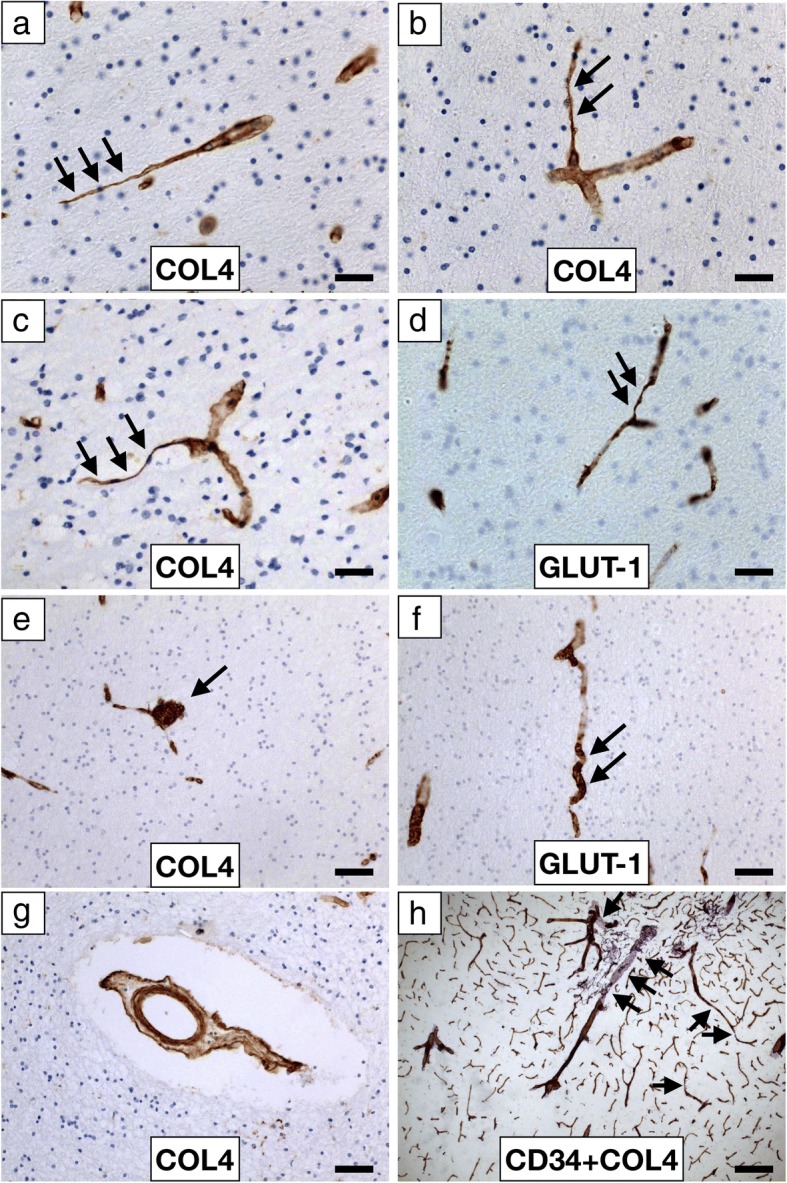
Microvascular pathology in the frontal WM in dementia **a-h**, Low and High power representative images of COL4 (**a**, **b**, **c**, **e**, **g**), GLUT-1 (**d**, **f**) and CD34 + COL4 (**h**) immunostained capillaries and microvessel in the WM. Collapsed and string vessels (arrows) were observed using both markers in VaD and PSD with similar profiles in all dementias. **e,** a microaneurysm-like structure (arrow) in a PSD case detected using COL4. **f**, a GLUT-1 immunmopostivie tortuous capillary (arrows) in AD. **g**, COL4 immunopositive ‘bagged’ vessel with increased perivascular space in a PSD case. **h**, CD34 and COL4 positive profiles of arterioles and capillaries at the juxtaposition of the grey and WM showing several collapsed and string capillaries (arrows). Scale bar represents 25 μm (**a**, **b**, **c** and **d**); 50 μm (**e**, **f**, and **g**); 100 μm (**h**)

Whole coronal sections at levels 6–8 [21, 31] containing the frontal lobe (Brodmann area 9) were immunohistochemically stained and analysed. Immunohistochemistry for collagen IV (COL4), a marker of basement membrane in the vessels and glucose transporter-1 (GLUT-1) or CD34, markers of endothelial cells were performed to assess various microvascular structures (Figs. 1 and 2). Tissue sections underwent antigen retrieval by using 12 min heating in a microwave oven with citrate buffer, pH 6.0 before being quenched with 3% hydrogen peroxide in Tris-buffered saline (TBS). Sections were then blocked with serum which was derived from the species in which the secondary antibody was generated, for 30 min. After the blocking processes, sections were treated with the primary antibodies against COL4 (1:1000, Sigma) and GLUT-1 (1:200, Thermo Scientific) or CD34 (1:1,000, Dako), 4 °C overnight followed by incubation with an appropriate secondary antibody (biotinylated anti-IgG; 1:200, Vector Laboratories, USA) for 30 min at room temperature. Visualisation for standard colour immunohistochemistry was performed using the Vectastain ABC System (Vector Laboratories) for 30 min at room temperature. After the final wash phase, the immunocomplexes were detected with diaminobenzidine (DAB). Again, at least two of both positive and negative controls were included to monitor the quality of staining.

### Assessment of microvascular density

**Fig. 3:**
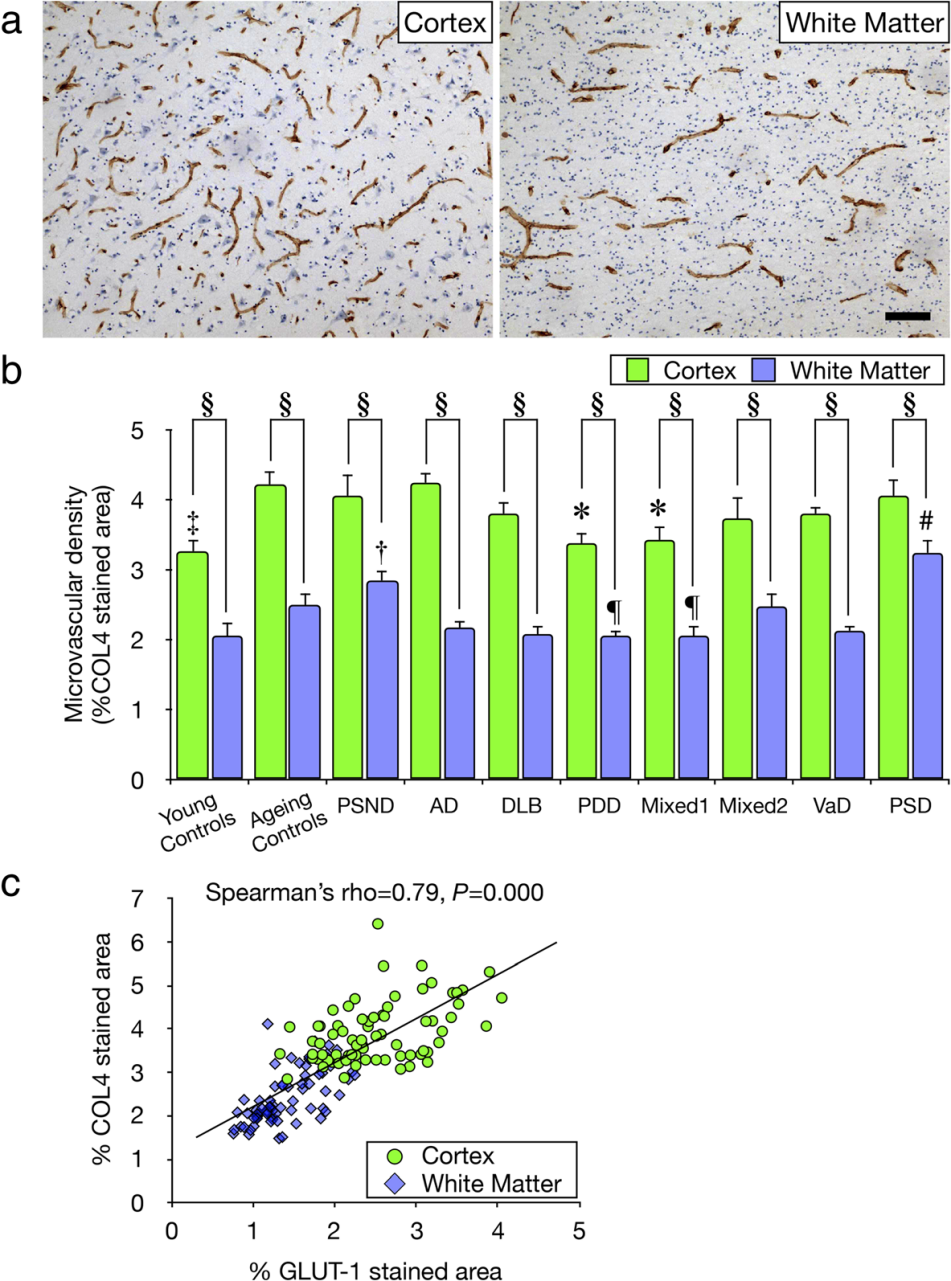
Quantification of microvascular density **a**, Typical images of COL4 immunostained capillaries in the cortex and WM used to quantify densities. Scale bar represents 50 μm. **b**, Histogram showing microvascular densities in the WM and cortex in controls and different dementias. In the WM, mean microvascular density was consistently lower by ~ 49% compared to cortex in all controls and dementia groups (^§^*P* < 0.01). In different dementias, microvascular density in the WM was decreased by ~ 18% compared to ageing controls, particularly in PDD and Mixed dementia 1 group (^¶^*P* < 0.05), whereas PSD and PSND showed ~ 57% higher microvascular density (^#^*P* < 0.012 vs all disease and control groups other than PSND; ^†^*P* < 0.025 vs all disease groups other than ageing controls, Mixed 2 and PSD). In the cortex, dementia subjects showed ~ 20% lower vascular density compared with ageing controls, particularly in the PDD and Mixed dementia 1 group (*P < 0.05). Young control group showed less vascular density by ~ 23% compared with ageing controls (^‡^*P* < 0.01). **c**, Correlation of microvascular densities between COL4 and GLUT-1 immunostained areas in dementia subjects. Spearman’s correlation analysis revealed a strong positive correlation (rho = 0.79, *P* = 0.000)

COL4 and GLUT-1 stained frontal lobe (Brodmann area 9) brain sections were analysed to assess microvascular, predominantly capillary, density. A total of ~ 7000 images of stained sections were collected prior to analysis. For each section, at least ten images from the entire depth of the cerebral cortex and the deep WM (removed from ventricular surfaces) were randomly captured using a bright field microscope (Leitz DIALUX 20, Leica) with a 10x objective lens coupled to an Infinity Capture2 camera running on Infinity Capture (Lumenera Corporation®) imaging software. Using image Pro Plus software (V.6.3, Media Cybernetics, Silver Spring, MD, USA), each image was traced and measured (pixels) to calculate percentage of COL4-stained or GLUT-1-stained area ((pixels/pixels)*100, %) as a proxy for microvascular density. We previously showed that estimation of microvascular density by stained pixels was correlated with manual count of microvessel length density (L_v_) profiles in serial sections [7]. Furthermore, vascular densities assessed by COL4 and GLUT-1 staining in the WM and cerebral cortex determined for each case were strongly correlated with each other (Fig. 3).

### Assessment of capillary width

Sections from the frontal (Brodmann 9) lobe were immunostained with COL4 and GLUT-1 (Brodmann area 9) and analysed to assess capillary widths. Capillaries were carefully identified by their width, suggesting distinct absence of myocytes. We previously established 3-dimenstional stereology and 2-dimensional (2D) methods were entirely consistent to quantify capillary widths [7]. Here, we used 2D imaging to quantify capillary widths from the immunostained sections containing the WM and overlying cortex. In total, we analysed over 684,000 capillary profiles in frontal lobe serial sections from 153 different dementia and control subjects. In most cases, we analysed 100–190 capillaries from each WM and cortical region. Longitudinally cut vessels were preferred for measurement (Fig. 1). A centre measurement with two other at the 1st and 4th quartiles were taken to create a representative measurement. Any unusually large (arteriole) or narrow vessel which appeared damaged was avoided, including string vessels and vessels in which a pericyte(s) was present. To make up to 100 profiles per case, occasionally transversely, cut vessels were measured in two dimensions and the mean diameter determined.

In preliminary experiments, we determined the best method to assess capillary width of diameter by comparing three different methods vis a vis the ‘VasCalc’ method [37], Image-Pro Analyser method and manual method. In the VasCalc method, the VasCalc software, developed previously to measure sclerotic index as well as diameters [37], was used to determine capillary width or diameter of longitudinal microvessel profiles in three different axes (Fig. 1). All width measurements were taken from 40x magnification images. For the Image-Pro Analyser, images taken at 10x magnification were used to make three manual measurements per capillary profile. The measurements then averaged to calculate mean capillary diameter by converted the pixel values to μm (*0.74). For the manual method, widths of capillary profiles in optically focused images taken at 25x magnification were measured using a micro graticule. The measured values in mm for each vessel were then converted to μm using the formula 2 mm = 1 μm. Overall, the three methods gave similar results. For example, the mean capillary widths in WM or the cortex calculated using the VasCalc software and Image-Pro Analyser methods were not significantly different (*P* = 0.176, Shapiro-Wilk for similar variances). The VasCalc was used as the method of choice to represent the results. Unless otherwise stated, at least 18 images per case were captured using Zeiss AxioPlan 2 microscope with Plan-Neuofluar® objectives using the Infinity Capture2 camera running on Infinity Capture (Lumenera Corporation®) imaging software.

### Statistical analysis

Statistical analysis was carried out using SPSS (IBM, version 23.0, IBM Corporation, Armonk, NY, USA) with the level of significance set at *P* < 0.05. First, distribution of values was tested using the Shapiro-Wilk test followed by one-way analysis of variance (one-way ANOVA) with post-hoc Tukey’s tests for normally distributed values or Kruskall-Wallis H tests for non-normally distributed values to compare data amongst dementia and control groups. Student’s t-test or Mann-Whitney U test was used for normally or non-normally distributed data respectively to assess the differences between cortex and WM data. Pearson’s correlation analysis was performed for assessing correlations between COL4 and GLUT-1 capillary width and the relationship between mean COL4 width of each of the disease groups and WM lesions derived from the vascular pathology scores [10], as the data was normally distributed. Spearman’s rho correlation analysis was used to assess correlations between COL4 and GLUT-1 vascular density, as the data was not normally distributed.

## Results

### Clinical and pathological features of the cases

Demographic details of the dementia subjects and controls are shown in Table 1. The mean age of subjects was not different between ageing controls and dementia groups, whereas young controls obviously younger than ageing controls and all dementia groups (*P < 0.01). The total CAMCOG and MMSE scores indicated all subjects had evidence of dementia at least 6 months prior to death. There were no differences in CAMCOG scores including memory and executive sub-scores and MMSE scores amongst dementia groups. We further noted that 55% of the dementia subjects exhibited hypertension (range 41–69% in each dementia group) and often had more than one other vascular disease risk factor including diabetes mellitus, ischaemic heart disease or smoking.

Neuropathological examination ensured appropriate classification of the AD and Mixed cases with high Braak and neuritic plaque scores. Lewy body counts and degree of neuronal loss in the substantia nigra were used to classify DLB and PDD cases. Notably, all dementias revealed significantly high vascular pathology scores compared to ageing controls (Table 1). The PSND subjects as expected also showed high vascular pathology scores. The WML scores were also significantly greater in all the dementias and even higher in VaD, PSD and Mixed 2 cases compared to ageing controls. Compared to ageing controls, all dementia groups also exhibited moderate to severe WM rarefaction and variable myelin loss with mean WML scores of > 2 (prevalence of moderate to severe white matter/vascular lesions) (Table 1).

### Microvascular pathology in the WM

Conventional staining and immunohistochemistry with COL4 and GLUT-1 antibodies revealed several abnormalities in microvessels of the deep WM across all dementias (Fig. 2). As implied by the vascular pathology scores (Table 1), we noted variable arteriolosclerosis, hyalinisation, myelin loss, microinfarcts and perivascular spacing with some vessels exhibiting fibroid necrosis and occasionally microaneurysms. We also found tortuous or coiled capillaries immunostained by both markers COL4 and GLUT-1. There were numerous collapsed or string microvessels with absence of GLUT-1 immunoreactivity; greater in some dementias than others (Fig. 2). Semi-quantitative grading indicated the severity of string capillaries or microvessels to be in the order: VaD = PSD > AD >Mixed 2 > Mixed 1 > DLB > PDD > > Controls. PSND had high number of string vessels but they were less than those in PSD cases.

### Microvascular density in the WM across the dementias

Quantification of COL4 immunostained microvessels showed microvascular density in the deep WM was lower by 20–49% compare to the entire depth of the frontal cortex across all dementias, PSND and controls (^**§**^*P* < 0.01) (Fig. 3a and b). The WM, except in PSD and PSND subjects, generally showed ~ 18% lower microvascular densities compared to ageing controls, particularly in PDD and Mixed 1 dementia subjects (^¶^*P* < 0.05). The PDD and Mixed 1 subjects also showed ~ 20% lower cortical microvascular densities compared to ageing controls (*P < 0.05) (Fig. 3b). In PSD, WM but not cortical microvascular densities were higher by 14% compared to those in PSND subjects. Microvascular density was greater by ~ 23% in the cortex in ageing controls compared to young controls (^**‡**^*P <* 0.01). We further demonstrated that microvascular densities were similarly altered retaining the trends in changes when GLUT-1 was used as the endothelial cell marker. Lower GLUT-1 densities were evident in the WM compared to cortex whereas the % GLUT-1 immunostained area in both the WM and cortex showed a strong positive correlation in dementia and ageing controls (Spearman’s rho = 0.79, *P* = 0.000) (Fig. 3c). In keeping with this calculation, the mean ratios of GLUT-1:COL4 in ageing controls in the WM was 0.8 whereas that in the cortex was 0.75 (*P* > 0.05). These ratios were not significantly changed in either VaD or AD subjects (data not shown).

### Capillary width in the WM in dementia

**Fig. 4:**
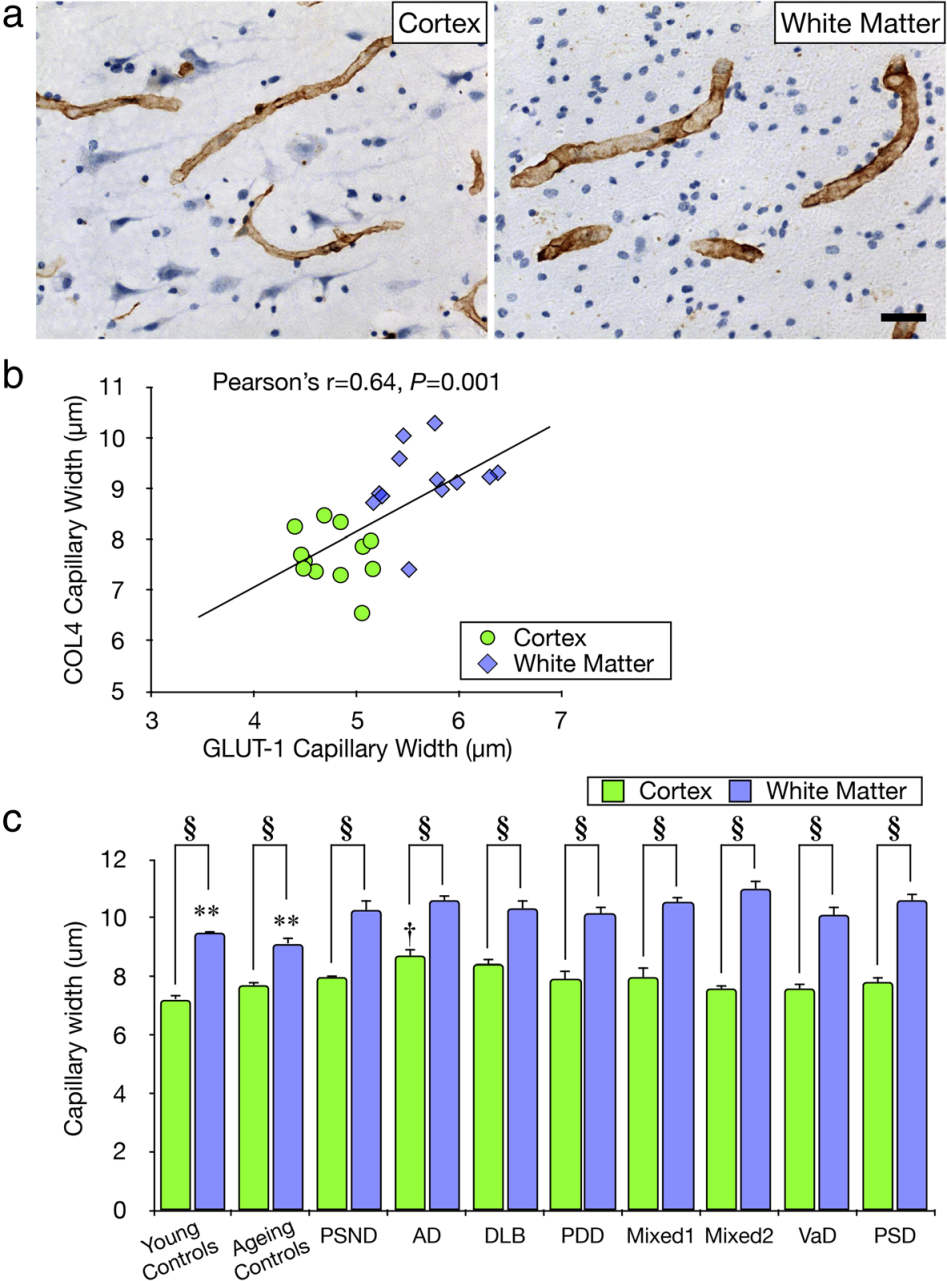
Quantification of capillary width **a**, Representative images of COL4 immunostained capillaries in the cortex and WM used to determine capillary width. Scale bar represents 25 μm. **b**, Correlation of mean capillary widths assessed by COL4 and GLUT-1 immunostaining in ageing controls. Pearson’s analysis revealed that mean capillary width in both the WM and the cortex was positively correlated (r = 0.64, *P* = 0.001). **c**, Histogram showing mean capillary width in the WM and cortex in controls and dementia groups. In the WM, mean capillary width was consistently larger by 19–45% compared to cortex in all control and dementia groups (^§^*P* < 0.01). In all the dementias, capillary width in the WM was consistently greater by < 20% compared to ageing and young controls (**P < 0.01). In the cortex, mean capillary widths in dementia subjects were not significantly wider compared with ageing and young controls, but only AD subjects showed wider capillaries compared with ageing and young controls (^†^*P* < 0.01)

We first quantified widths of frontal WM and cortical capillaries labelled by COL4 and GLUT-1 immunoreactivities in control subjects (Fig. 4). We found that mean capillary widths assessed by COL4 and GLUT-1 were positively correlated in the cortex and the WM (Pearson’s r = 0.64, *P* = 0.001) (Fig. 4b). The dot plot  indicated the capillary widths in the WM were greater compared to the cortex. In further quantitative analysis of COL4 immunostained capillaries across all dementias and controls, we confirmed that the WM capillaries exhibited wider widths by ~ 31% compared to the cortex in all subjects (^**§**^*P* < 0.01) (Fig. 4a and c). However, surprisingly we found that the capillaries in the WM were marginally but significantly wider (**P < 0.01) in all dementias irrespective of type compared to ageing and young controls (Fig. 4c). In the cortex, the capillaries in dementia subjects were not significantly wider compared with the controls, but AD group showed significantly wider capillaries compared to controls (^**†**^*P* < 0.01) (Fig. 4c).

**Fig. 5:**
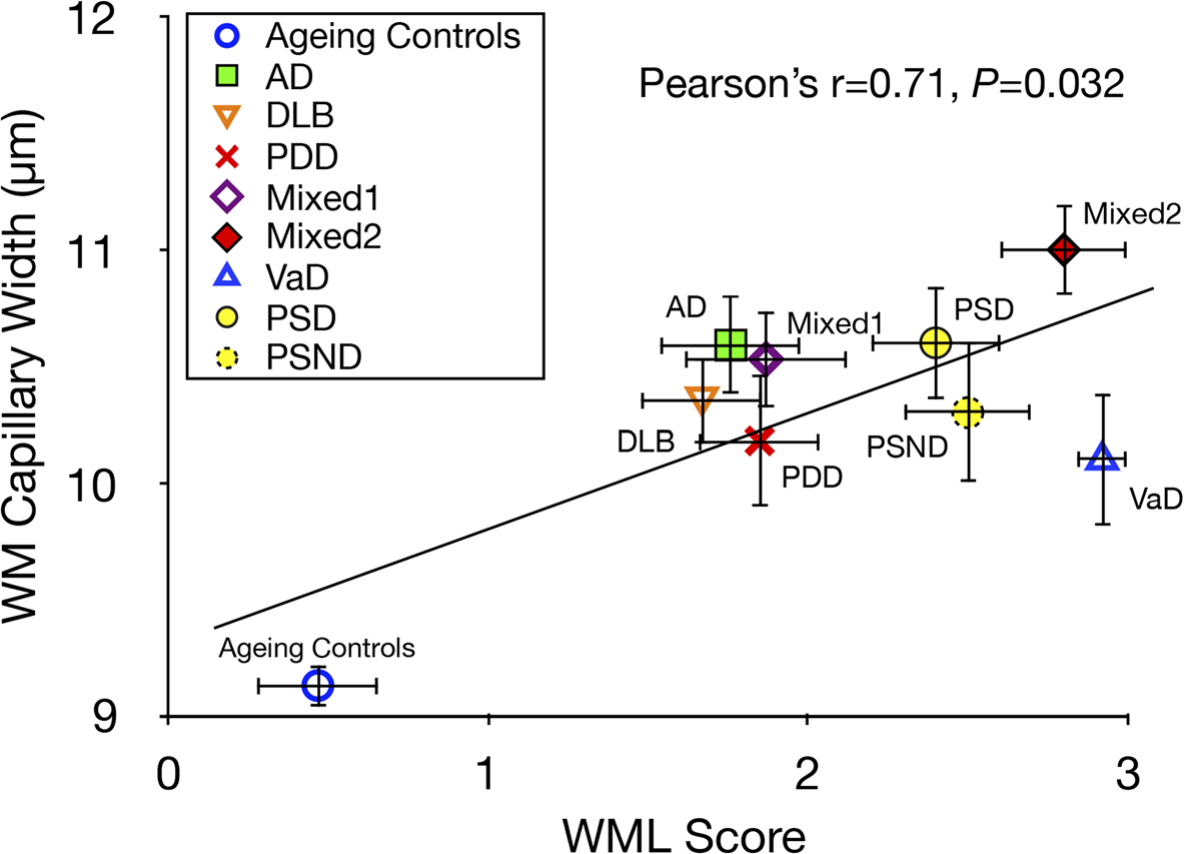
Relationship between WM capillary width and WM pathology Plot shows correlation between mean capillary widths assessed by COL4 immunostaining in all the dementias and ageing controls versus WML scores. Pearson’s analysis revealed that mean capillary width in the WM in dementia was positively correlated with WM damage (r = 0.71, *P* = 0.032). Although there were some age-related changes in WM in the controls, it was clear that all dementias were a disparate group as a whole exhibiting high WML scores and wider capillaries

To test whether WM capillary changes were related to ensuing WM pathology, we plotted mean values of capillary widths from different dementia groups and ageing controls against WML scores (Fig. 5). We found a moderate correlation indicating greater capillary width was associated with greater WML scores (Pearson’s r = 0.71, *P* = 0.032).

## Discussion

Analyses of different dementias using repeated robust methods of quantification in a large series of samples indicate several novel observations related to microvessels, largely capillaries of the WM compared to those of the cortex in ageing brain. We first report high degree of SVD pathology and capillary abnormalities in the WM in clinically assessed subjects with different neurodegenerative dementia diagnoses including DLB, PDD, Mixed dementias and AD. This was corroborated by the vascular pathology as well as WML scores. Whereas the highest burden of SVD pathology [29] consisting of severe arteriolosclerosis, WM rarefaction, microinfarction and perivascular spacing was present in VaD and PSD, it was intriguing that subjects diagnosed with neurodegenerative dementias including PDD, DLB and AD exhibited similar SVD changes. Arteriolosclerosis and microinfarcts, which are strongly associated with cognitive impairment [2, 20, 36], were consistent features in different dementia types. Capillary abnormalities including tortuous or coiled capillaries as well as collapsed string vessels were evident particularly in VaD and PSD cases, suggesting cerebral hypoperfusion or ischaemic changes are likely the cause of microvascular abnormalities. While these microscopic lesions are not apparent on T2-weighted  or FLAIR sequences from MRI [8, 9], they demonstrate they are present in tissue and may contribute to overall dementia diagnoses.

Quantification of microvascular pathology in the WM across the neurodegenerative dementias showed total COL4 immunopositive microvascular densities tended to be decreased by ~ 18% although they were increased by 52% in PSD compared to VaD subjects. We suggest the SVD type of pathological changes, particularly in the vascular dementias, reflects restructuring of the microvascular network by increasing capillary bed to counter hypoperfusion in the WM. Our results also suggest that subjects with more chronic state disease e.g. VaD versus PSD exhibit lower degrees of microvascular plasticity. When capillaries lose their endothelium by chronic cerebral hypoperfusion or ischaemic insults, the basement membrane is not degraded and form functionally useless string vessels that can no longer transport cells or plasma [6]. Previous results show that 3 to 5 days after ischaemic insults, capillaries lose endothelial cells presenting accordion-like pleating of residual basement membrane and leading to thin acellular strands at 40 days post insult. Since some string vessels were found in ageing controls although to a much lesser extent than in all dementias with extracellular pathology [6, 23], it suggests nominal vascular remodeling or restructuring also occurs in normal ageing brain. String vessel remnants can provide the skeletal structure for newly forming capillaries by invading capillaries forming new basement membrane within the old, presenting a duplicated basement membrane [6]. The basement membrane provides the supporting structure of a microvessel composed of several thin layers of insoluble extracellular proteins including collagen (50%) and the framework for growth of endothelial cells [30] and restoring the gliovascular unit [16].

Our results showed that both in controls (physiological) and pathological conditions, capillary width was consistently larger by ~ 45% whereas microvascular density was lower by ~ 49% in the WM compared to the cortex. In other words, capillaries in the WM were wider and sparse, whereas capillaries in the cortex were narrower and dense. These observations were verified by a correlation analysis of the two markers, COL4 as well as GLUT-1. The strong positive correlation between microvascular width assessed by COL4 and GLUT-1 confirmed that the size of entire capillary including the vascular lumen is larger in the WM compared to the cortex and this was increased by ~ 20% in dementia states. The robustness of these observations was strengthened by our previous studies [7] reporting strong correlation between COL4 and GLUT-1 immunostained microvascular length densities (L_v_) in the hippocampus (r^2^ = 0.687, *P* = 0.000). We similarly noted that capillary widths in the WM of the temporal lobe were larger by 17–20% than those in the overlying cortex (*P* < 0.01) and in dementia subjects including VaD, Mixed and AD (*P* < 0.01) compared to controls (unpublished results). The WM versus cortical or gray matter differences in microvascular densities [25] and capillary sizes appear specific and likely reflect cellular contents of the regions. Protection of neurons in the cortex requires greater supply of oxygen and nutrients reflecting higher microvascular densities whereas blood flow per unit length of WM capillary is increased. While similar conclusions may be made for GLUT-1 results as those for COL4, these novel findings with respect to dementia suggest there are compensatory mechanisms in the WM to maintain reserves of blood flow within capillaries and ameliorate cerebral hypoperfusion [11, 16]. It is plausible that capillary dilation with the reflected structural modifications leads to a local increase in the number of erythrocytes of ~ 6 μm in diameter travelling in a single file [33] as an adaptive mechanism to increase oxygen supply in the chronically hypoxic deep WM [12]. That WM capillaries also tended to be increased in width in the PSND subjects suggested it was the presence of vascular pathology in the WM that likely instigates widening of the capillaries. However, we cannot refute that the wider vascular width in the cortex evident in AD or Mixed dementia subjects could be due to microangiopathy attributed to amyloid or other proteins, particularly adhering to capillaries [19].

We emphasise that one of the main limitations of our study is that brain regions other than the frontal lobe were not systematically investigated for SVD pathology. We also did not quantify the densities of string or coiled vessels across all dementias. This is an extremely cumbersome undertaking and we deemed it would not improve the outcomes over the semi-quantitative results presented. While we concentrated on the deep WM of the frontal lobe in accord with our prior hypothesis [16], we had previously demonstrated the spectrum of SVD pathology in different dementias including VaD, AD and DLB incorporating the temporal lobe and the basal ganglia [10]. Given that the same neurodegenerative pathologies occur in PDD and other mixed dementias, it is reasonable to suggest similar microvascular or capillary changes occur in the WM of these dementias. Quantification of capillary widths in more regions of the brain can also be quite cumbersome. Still we deem such an undertaking in the future would inform on the relative degrees of microvascular abnormalities in different dementias, as assessment of microvascular abnormalities potentially distinguish dementias with more severe vascular insults (such as VaD and PSD) from other neurodegenerative dementias.

## Conclusions

In summary, we provide evidence for widespread microvascular pathology in the frontal WM relative to the cortex in neurodegenerative as well as dementias caused by vascular disease. We also showed that capillaries of the deep WM have greater diameters compared to the overlying neocortex and that capillary width sizes are increased in different dementias. Our results imply chronic hypoperfusion induces microvascular modification or restructuring in the deep WM that may affect the function of the gliovascular unit and WM perfusion.
